# Multifaceted Evaluation of Ultra-high-field 9.4-T Magnetic Resonance Imaging after Inorganic Tattoos: An Animal Study

**DOI:** 10.31662/jmaj.2019-0012

**Published:** 2019-06-06

**Authors:** Shoichi Tomita, Takeshi Miyawaki

**Affiliations:** 1 Department of Plastic and Reconstructive Surgery, JCHO Tokyo Shinjyuku Medical Center, Tokyo, Japan; 2 Department of Plastic and Reconstructive Surgery, The Jikei University School of Medicine, Tokyo, Japan

**Keywords:** Magnetic resonance imaging, burn, tattoo, permanent makeup

## Abstract

**Introduction:**

Tattooing techniques for reconstruction and rectification of the sequelae of cancer treatments, surgery, and scarring have received attention in the medical field. There is concern that tattooed skin is at risk of being burned by magnetic resonance imaging (MRI) examination. However, a detailed evaluation of the impact of MRI imaging on medically tattooed skin has not yet been performed. This study aimed to clarify the changes in common inorganic tattoo pigments during MRI examinations in an animal model.

**Methods:**

Tattooed hairless mice with eight typical pigments for tattoos were evaluated for changes in temperature, color tone, and histology of the tattoos during a 9.4-T MRI.

**Results:**

None of specimens had signs of burns, such as redness or blisters. In terms of temperature changes, the black iron oxide and carbon black specimens had a maximum temperature increase of 0.4 degrees. In the Munsell color system, no specific color changes were observed before or after the MRI. Color changes, evaluated as the ΔE_00_ in the L^*^a^*^b^*^ color space, were all below 3.0 and were thus regarded as being indistinguishable and within the color unevenness of the tattoo. Histologic analysis of the specimens showed no significant changes before and after the MRIs.

**Conclusions:**

The observed temperature changes, color tone changes, and histological changes in the tattooed areas in this animal model were not of a magnitude considered likely to adversely affect the human body.

## Introduction

In recent years, tattooing has been utilized for beautification, via procedures called permanent makeup, dermapigmentation, micropigmentation, or cosmetic tattooing. Tattooing techniques have gained popularity in the medical field, with paramedical pigmentation being used for procedures such as the reconstruction of the nipple-areola complex in breast cancer, rectification of eyebrow hair loss during chemotherapy, and improvement of vitiligo and white scars. An estimated 24% of Americans aged between 18 and 50 years ^(1)^, and 26% of male and 20% of female Australians aged between 19 and 23 years ^(2)^, have tattoos. In these two countries, licensed or qualified artists create the tattoos. In contrast, in Japan legal tattooing or permanent makeup procedures are considered medical practice. The United States Food and Drug Administration does not approve pigment injection into the body, and approved ingredients are only used for cosmetic purposes ^(3)^. Therefore, ensuring of safety is required.

There are reports of adverse events, including pain, swelling, burning sensation, and burns during magnetic resonance imaging (MRI) in patients with tattoos or permanent makeup. The first report describes a 22-year-old woman with first-degree burns in the area of the permanent eyeliner following head and cervical vertebrae MRI ^(4)^. Subsequently, Siemens (Siemens, Bayern, Germany) discouraged patients with permanent eyeliners from undergoing MRI examinations. There have been five subsequent cases in which burning sensation or redness or swelling, has been reported to be associated with permanent make-up ^(5), (6), (7), (8)^, with short recovery times, and seven cases of burns related to MRI in tattooed areas ^(10), (10), (11), (12), (13), (14), (15)^. Only one case of a second-degree burn has been reported ^(11)^.

Manufacturers do not recommend MRI examinations for patients with tattoos or permanent make-up ^(4)^. However, in cases where MRI is considered necessary, the procedure is conducted with sufficient informed consent. For example, tattoo for nipple-areola complex after breast reconstruction is one of the major tattoos performed in our clinic. Those patients often have MRI for breast cancer follow-up. This approach varies among hospitals and doctors in charge, and currently there is no consensus ^(16)^.

Therefore, the present study aimed to clarify the change that takes place in common inorganic tattoo pigments that are mainly used for nipple-areola tattoo post MRI using an animal model.

## Materials and Methods

The protocol for the animal experiments was reviewed and approved by the Institutional Animal Care and Use Committee of the Jikei University (No. 2015-053) and conformed to the Guidelines for the Proper Conduct of Animal Experiments of the Science Council of Japan (2006).

### Pigments

From 30 colors (Pure Pigments, Biotouch, Inc., CA, USA), the following eight basic ingredients were selected: titanium dioxide; iron oxide yellow; iron oxide red; iron oxide black; carbon black; blue ultramarine blue #29; green chromium oxide green #7; and D&C red #7. Pigments were created by mixing these ingredients.

### Tattooing procedure

**Figure 1.**
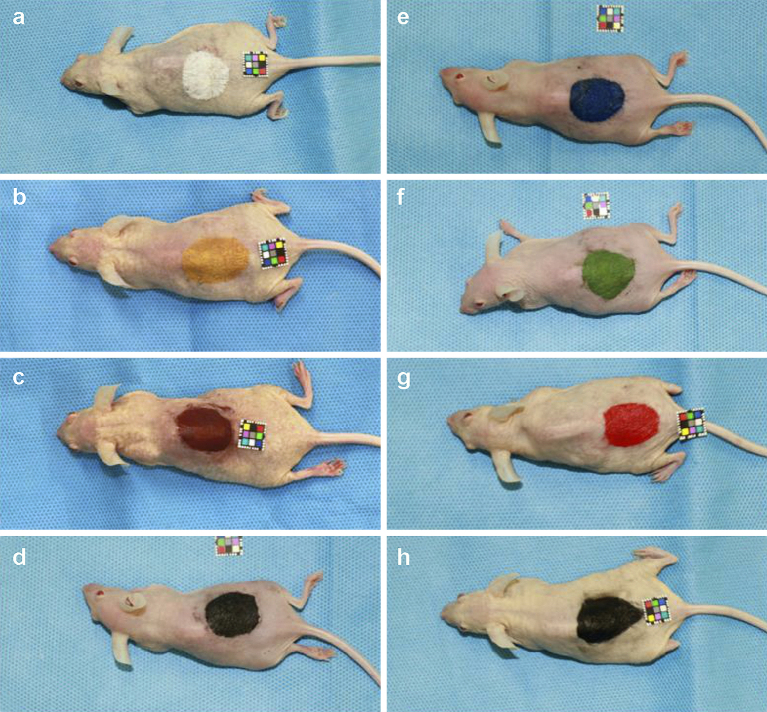
Mice tattooed with pigments containing metal components. A 25-mm circular tattoo was applied to the back of hairless Hos:HR-1 mice. The utilized colors were: (a) titanium dioxide; (b) iron oxide yellow; (c) iron oxide red; (d) iron oxide black; (e) ultramarine blue #29; (f) chromium oxide green #7; (g) D&C red #7; and (h) carbon black.

Under general anesthesia (2.5% isoflurane), a circular tattoo for each selected color pigment (25mm in diameter) was applied to the backs of eight hairless Hos:HR-1 mice, and petroleum jelly was subsequently applied before they were awakened (Figure 1). Additionally, as a control, one mouse was treated with needles, but without pigments. We decided on this tattoo size being similar to the size of the areola.

### MRI examinations

**Figure 2.**
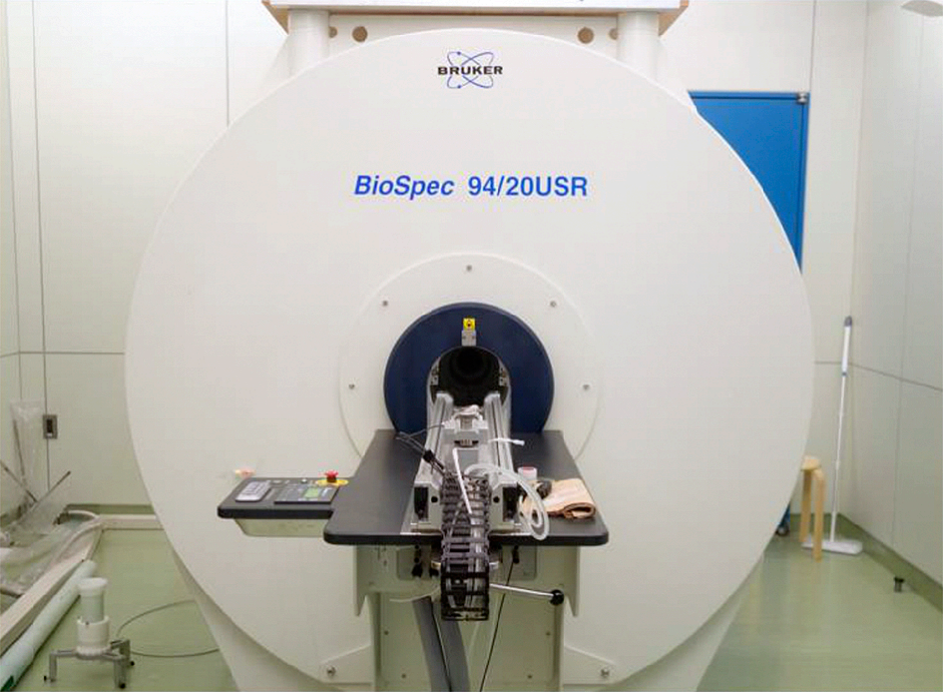
An ultra-high magnetic field magnetic resonance scanner (9.4-T) manufactured by Bruker (BioSpec 94/20 USR) was used for magnetic resonance imaging. The sequence was as follows: repetition time 3,500 msec; Echo time 11.0 msec; Flip angle, 180°.

**Figure 3.**
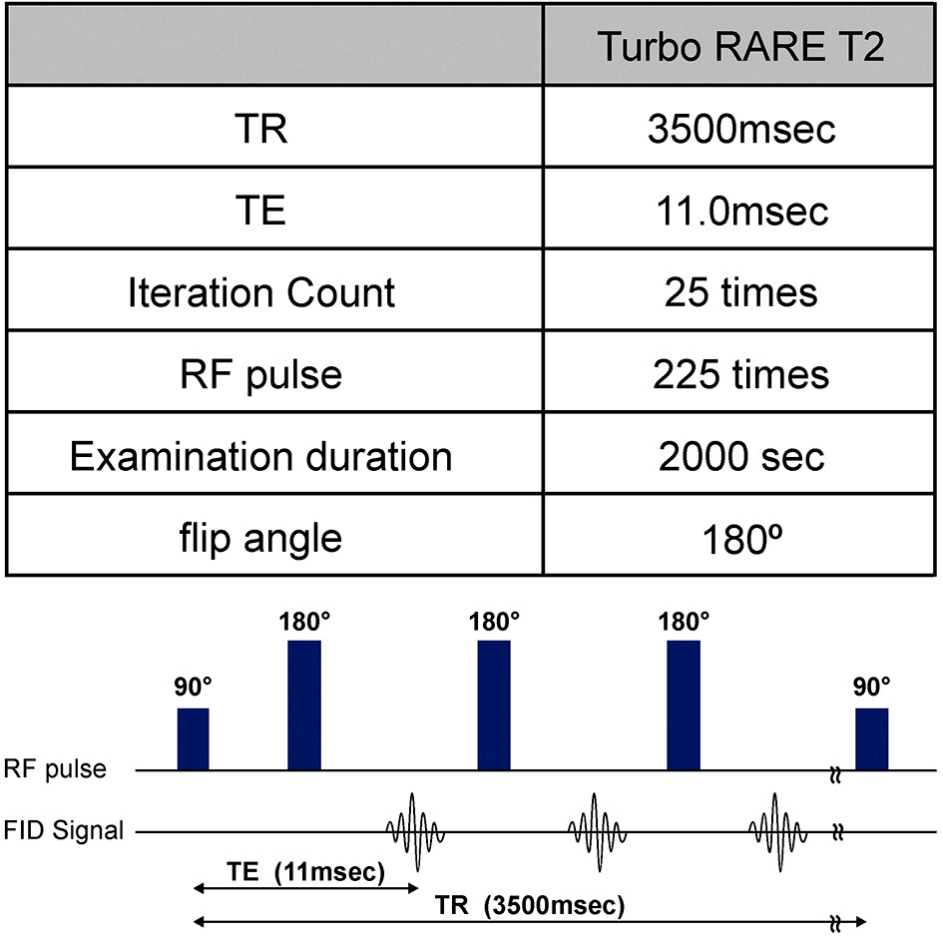
Sequence of the magnetic resonance imaging (MRI). The spin echo method used to obtain a radiofrequency (RF) pulse echo was the Turbo RARE T2 sequence.

The day after tattoo application, MRIs were conducted on all mice using an ultra-high-field 9.4-T machine (Bruker BioSpec 94/20 USR, TX, USA) (Figure 2). Twenty-five consecutive MRIs were performed, twice on each color, using the Turbo RARE T2 sequence (Repetition time 3500 msec; Echo time 11.0 msec; flip angle, 180°), which is a spin echo method with many radiofrequency (RF) pulses (Figure 3).

The four evaluated variables were: (1) change in tattooed area-surface temperature during MRI; (2) presence or absence of burns post MRI; (3) difference in color tones of tattoos before and after the MRI (determined using a spectrophotometer); and (4) differences in the histological characteristics of the tattooed area before and after MRI.

### Temperature assessments

**Figure 4.**
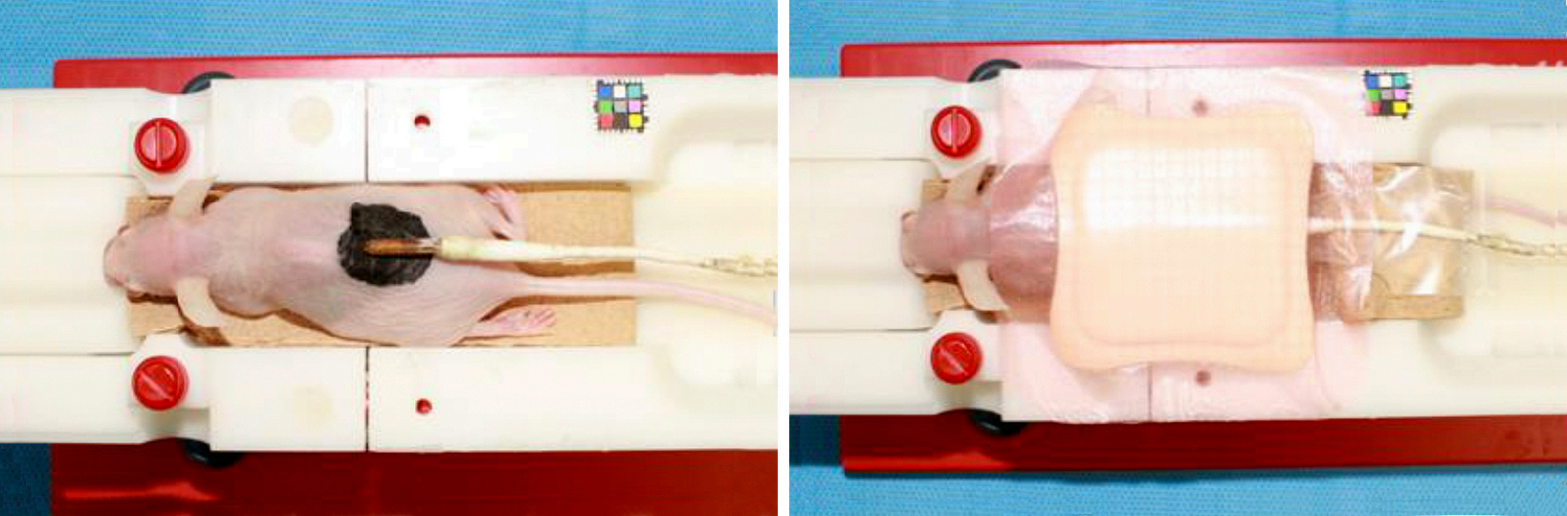
Surface temperature measurement during the magnetic resonance imaging procedure. The body temperature probe was applied to the tattooed area, which was insulated on one side using polyurethane foam. The animal was placed in the gantry of the MRI scanner (Figure 2) for more than 30 min; when the temperature measurement plateaued, the MRI began.

Temperature changes during the MRIs were determined using a body temperature probe on the tattooed areas, with polyurethane foam on one side for thermal insulation (Figure 4). Additionally, temperature changes on the biological monitor were continuously recorded. Although the internal temperature of the MRI examination room was maintained constant via air conditioning, the temperature inside the MRI apparatus was slightly lowered by the cooling system. Thus, the mouse was placed in the gantry for 30 min or longer; when the temperature stabilized, imaging was started.

### Color analysis

Color analysis of the tattooed area before and after MRI was performed using a spectrophotometer (CM 600d Konica Minolta Japan Co., Ltd.) and color management software (CM-S100w SpectroMagic NX Konica Minolta Japan Co., Ltd.). The color tone in each tattooed area was measured 10 times and the mean was calculated. Data were recorded as mean ± standard deviation. We used (1) the Munsell color space ^(17)^; and (2) the color space defined by the Commission International de L’eclairage, which is based on one channel for luminance (lightness) (L) and two channels for color (a and b) (L^*^a^*^b^*^) ^(18)^. The three attributes in the Munsell color system are hue (H), value (V), and chroma (C). H reflects the name of any color, as found in its pure state in the spectrum, which is divided into five principal groups: red; yellow; blue; green; and purple. Additionally, yellow-red, green-yellow, blue-green, purple-blue, and red-purple are five intermediate hues. These hues are represented by numbers up to 10. This numerical value indicates the lightness/darkness of the color, with darker colors closer to 0 and brighter colors closer to 10. The chroma indicates color vividness; colorless and achromatic color has a chroma of 0, and the chroma increases as the color vividness increases. However, the maximum value of the chroma differs, depending on the hue and value. Achromatic color is specified as N V ^(17)^.

In contrast, the L^*^a^*^b^*^ color space is a uniform color space that can evenly display color differences as perceived by the human eyes. L^*^ represents the psychometric lightness and refers to the brightness; greater values (from 0 to 100) reflect brighter colors. The symbols a^*^ and b^*^ are called the chromatic indices and reflect the direction (hue/saturation) of a color in the color space. The further a^*^ is in the positive direction, the redder the color; conversely, the further a^*^ is in the negative direction, the greener the color (the complementary color to red). The further b^*^ is in the positive direction, the more yellow the color; conversely, the further b^*^ is in the negative direction, the bluer the color (the complementary color to yellow) ^(18)^. The L^*^a^*^b^*^ color space is used to evaluate color changes (i.e., the color difference, ΔE_00_). Two colors with a ΔE_00_ of 0.8–1.6 are classified as having grade AA color tolerance; the difference in color can be recognized if samples are placed side-by-side. A ΔE_00_ of 1.6–3.2 indicates grade A tolerance; the colors are regarded as being indistinguishable and are generally considered the same color ^(19)^.

### Histologic analysis

Three-millimeter punch biopsy specimens were obtained from the tattooed areas before and after the MRI. The biopsy wounds were closed with 5-0 nylon sutures. Biopsy samples were immediately fixed in 10% formalin and embedded in paraffin. Vertical sections were obtained and stained with hematoxylin and eosin staining and blindly analyzed by a histopathologist.

## Results

### Temperature changes

**Figure 5.**
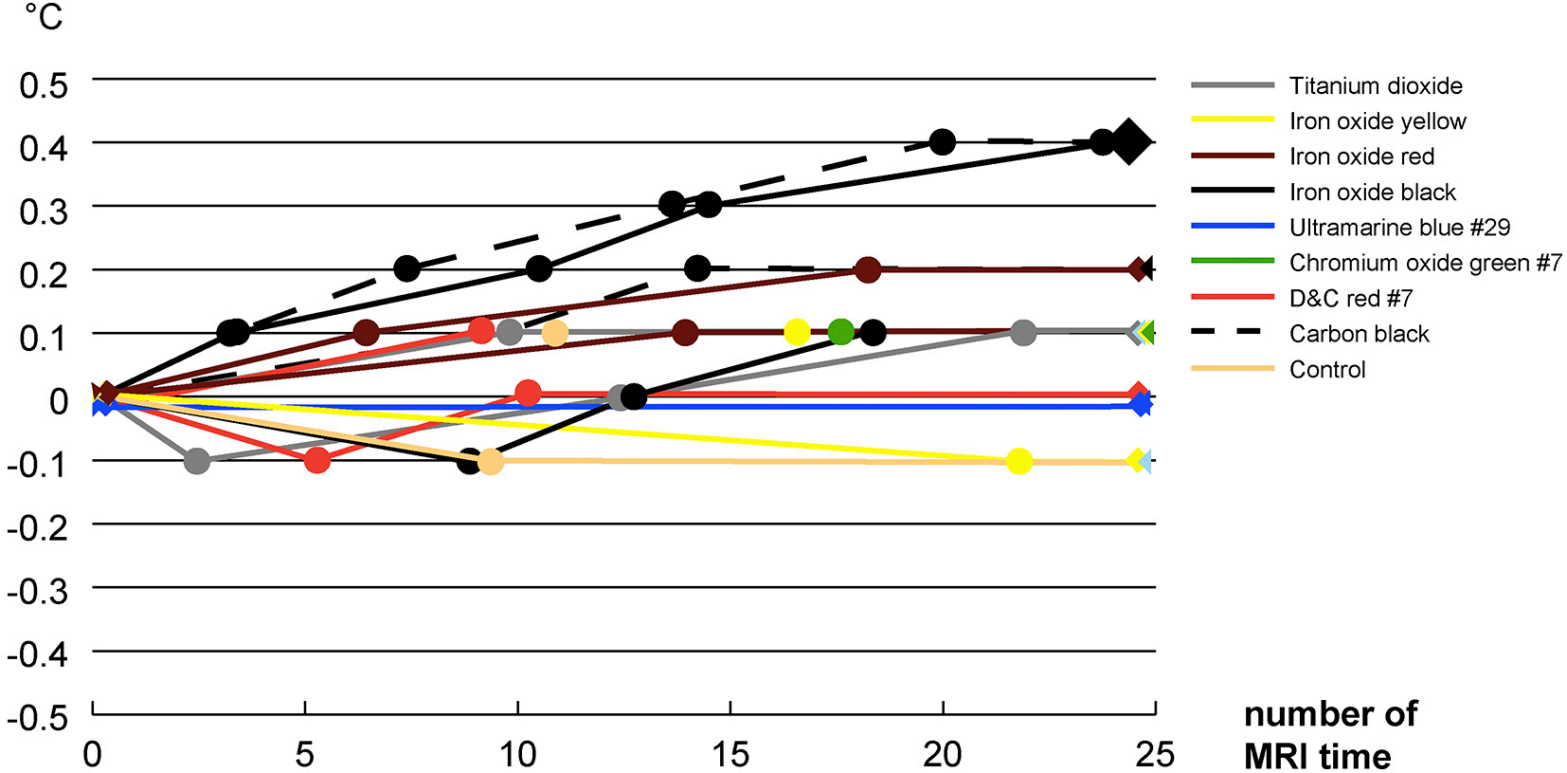
Temperature changes during the magnetic resonance imaging procedure. Temperature changes on the biological monitor of the MRI operation room were recorded continuously.

There was no change in temperature for blueish ultramarine blue #29. The control specimens, white titanium dioxide, yellowish iron oxide yellow, greener chromium oxide green #7, and reddish D&C red #7 had a maximum temperature increase of 0.1 degrees. The rusty iron oxide red specimen had a maximum temperature increase of 0.2 degrees. Black iron oxide and carbon black specimens had a maximum temperature increase of 0.4 degrees (Figure 5).

### Presence or absence of burns

None of the specimens had recognizable findings of burns, such as redness or blistering, on visual inspection.

### Color changes

Detailed results are presented in Table 1. Because the chroma for titanium dioxide was large (before MRI: 1.30 ± 0.11, after MRI: 1.25 ± 0.02), this indicated that the hue and chroma are chromatic. On the other hand, the chroma for iron oxide black and carbon black before and after the MRI was very small, less than 0.15, and was considered achromatic.

**Table 1.** Color Tone Changes before and after the MRI Examinations.

		Hue	Value	Chroma	L*	a*	b*	⊿E00
Titanium dioxide	Before MRI	1.2 ± 0.9 Y	6.10 ± 0.25	1.30 ± 0.11	62.51 ± 2.50	0.82 ± 0.40	9.41 ± 0.93	
								0.86
	After MRI	1.2 ± 0.2 Y	6.01 ± 0.01	1.25 ± 0.02	61.57 ± 0.11	0.85 ± 0.09	8.98 ± 0.05	
Iron oxide yellow	Before MRI	9.3 ± 0.0 YR	5.60 ± 0.01	5.51 ± 0.00	56.51 ± 0.11	12.94 ± 0.01	32.90 ± 0.02	
								3.00
	After MRI	9.4 ± 0.1 YR	5.93 ± 0.06	5.32 ± 0.04	59.77 ± 0.66	12.16 ± 0.06	31.64 ± 0.37	
Iron oxide red	Before MRI	7.7 ± 0.1 R	3.10 ± 0.01	3.51 ± 0.02	31.37 ± 0.14	16.63 ± 0.11	9.51 ± 0.09	
								0.38
	After MRI	7.3 ± 0.1 R	3.13 ± 0.02	3.46 ± 0.01	31.69 ± 0.18	16.49 ± 0.03	9.09 ± 0.03	
Iron oxide black	Before MRI		3.32 ± 0.10		25.22 ± 0.54	0.65 ± 0.06	0.19 ± 0.14	
								1.11
	After MRI		3.10 ± 0.05		26.34 ± 1.11	0.31 ± 0.25	−0.37 ± 0.40	
Ultramarine blue #29	Before MRI	6.1 ± 0.1 PB	2.49 ± 0.13	1.30 ± 0.09	25.96 ± 1.34	−0.70 ± 0.06	−5.81 ± 0.37	
								2.63
	After MRI	5.7 ± 0.1 PB	2.64 ± 0.01	2.01 ± 0.02	27.57 ± 0.12	−1.08 ± 0.03	−8.88 ± 0.07	
Chromium oxide green #7	Before MRI	7.0 ± 0.5 GY	4.14 ± 0.13	2.53 ± 0.29	42.42 ± 1.43	−8.46 ± 1.18	11.88 ± 0.88	
								1.35
	After MRI	7.7 ± 0.2 GY	4.24 ± 0.03	2.74 ± 0.09	43.37 ± 0.33	−9.62 ± 0.37	12.14 ± 0.27	
D&C red #7	Before MRI	5.4 ± 0.1 R	4.18 ± 0.09	8.11 ± 0.25	42.45 ± 0.97	33.32 ± 1.11	17.81 ± 0.37	
								0.87
	After MRI	5.3 ± 0.3 R	4.11 ± 0.05	8.45 ± 0.08	41.75 ± 0.53	34.79 ± 0.26	18.20 ± 0.59	
Carbon black	Before MRI		3.32 ± 0.10		34.12 ± 1.00	0.38 ± 0.03	0.34 ± 0.12	
								1.91
	After MRI		3.10 ± 0.05		31.78 ± 0.57	0.33 ± 0.04	0.67 ± 0.09	
Control	Before MRI	2.4 ± 0.1 YR	5.87 ± 0.05	2.13 ± 0.03	60.29 ± 0.48	5.59 ± 0.13	9.56 ± 0.08	
								0.90
	After MRI	3.3 ± 0.6 YR	5.79 ± 0.09	2.03 ± 0.11	59.69 ± 0.78	5.19 ± 0.44	10.14 ± 0.76	

Each color change is described in Munsell color space (hue, value and Chroma) and L^*^a^*^b^*^ color space (L^*^, a^*^ and b^*^). Color tone in each tattooed area was measured 10 times with a spectrophotometer and the average value was presented as mean ± standard deviation. Abbreviations: Y, yellow; YR, yellow-red; R, red; PB, purple-blue; GY, green-yellow (in the Munsell color space);

The maximum color change, ΔE_00_, was measured as 3.00 using iron oxide yellow. The H, V, and C were 9.3±0.0 YR, 5.60±0.01, and 5.51±0.00, respectively, before the MRI, and were 9.4±0.1 YR, 5.93±0.06, and 5.32±0.04, respectively, after the MRI. The L^*^, a^*^, and b^*^ were 56.61±0.11, 12.94±0.01, and 32.90±0.02, respectively, before the MRI, and were 59.77±0.66, 12.16±0.06, and 31.64±0.37, respectively, after the MRI.

With consideration of the range of the color unevenness of the tattoo, the ΔE_00_ (before vs. after the MRI) was less than 3.2 (grade A color tolerance) for all tattoos, i.e., the colors were regarded as indistinguishable and were considered to be the same general color. Moreover, using the Munsell color system, no characteristic changes in H, V, or C were observed (Table 1). Thus, we can say that there were no apparent differences in the colors of the tattoos before and after the MRI.

### Histological changes

**Figure 6.**
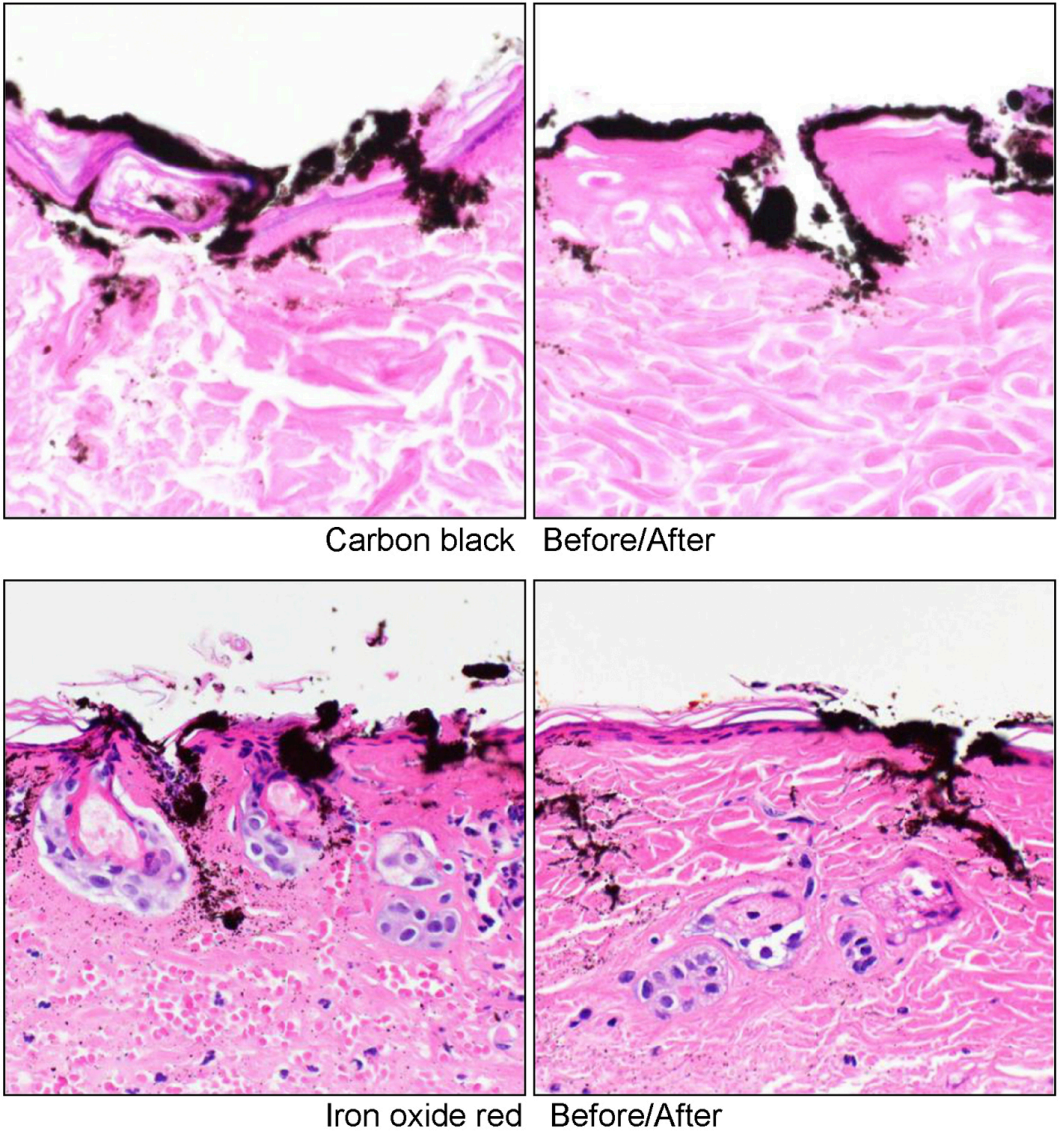
Tissue specimens before and after the magnetic resonance imaging examination. The pigments were found not only in the dermis, but also in the epidermis. It was confirmed that there was erosion of the epidermis. Neutrophil infiltration and the denaturation of collagen fibers were observed in the dermal layer.

In all specimens, pigments were found not only in the dermis, but also in the epidermis. It was confirmed that there was erosion of the epidermis, which was considered to be due to the tattooing treatment. Neutrophil infiltration and the denaturation of collagen fibers were observed in the dermal layer. However, no remarkable differences were observed between specimens taken before and after the MRIs; the histological findings were considered to reflect the surgical influence of the tattooing treatment (Figure 6).

## Discussion

This is the first study that clarifies the changes in common tattooing pigments in MRIs using an animal model. The three causes of burns during MRI examinations are: (1) electromagnetic induction; (2) resonance circuits; and (3) an antenna effect ^(20)^. Basically, when the induced current (Eddy current) generated by the electromagnetic induction of the MRI scanner causes Joule heating, the conductor generates heat. Factors causing electromagnetic induction include changes in the gradient magnetic field and RF irradiation, but the influence by the former is minor, and the influence by the latter is considered to be the main factor. The Joule heat generated in the human body by the RF pulse is evaluated using the heat absorption rate per unit (specific absorption rate). The International Electrotechnical Commission has set an upper limit of 0.4 W/kg for the entire body, 3.2 W/kg for the head, and 8.0 W/kg per 1 g of body tissue ^(21)^. Additionally, the increase in body temperature during an MRI must not exceed 1℃ and must not exceed 38℃ for the head temperature, 39℃ for the trunk temperature, and 40℃ for the extremities ^(20)^. Despite using an MRI scanner with a much stronger magnetic field (9.4 T) in the present study than that used clinically (1.5 T and 3.0 T), the maximum temperature increase in the tattooed areas with various pigments was 0.4 degrees. Therefore, the results suggest that the examination can be conducted safely.

The maximum temperature increases were in the iron oxide black (which is attracted to the magnet) and carbon black (which is not attracted to the magnet) tattooed areas. A previous study by Muranaka et al. ^(22)^ reported that heat generation accompanying RF irradiation occurs, even with a weak magnetic substance, and ferromagnetic substances tend to rise in temperature. In contrast, our results suggested that the heat generated by the RF pulse had little relation to the magnetism effect. Thus, not only is the presence of metal an important factor, but its shape must also be considered. Circuits formed at the time of the MRI examination are often caused by contact between a cable, such as an electrocardiogram monitor, and the skin, or by contact between a body and the bore (the cylindrical part of the scanner into which a subject enters). There are also reports of burn injuries due to circuits formed in the human body, such as when the hands are clasped on the head, when the hand is set on the waist, or when the legs are crossed, and so on ^(20)^. We believe additional studies on the temperature difference associated with tattoo shapes are required.

One of our major evaluated variables was the difference in the color tone of the tattoo measured by spectrophotometer before and after the MRI examination. Our study identified a color difference, ΔE_00_, before and after the MRI of less than 3.0, and thus regarded ΔE_00_ as being indistinguishable from the redness involved with heat. This was the result that supported the temperature assessments mentioned above.

Another interesting finding was the histological findings. Huzaira and Anderson ^(23)^ in their study showed that tattooed ferromagnetic pigment in the skin moved when a 1.4-T strong permanent magnet was attached to the tattooed area after Q-switched ruby laser treatment. However, our study failed to reveal histological changes associated with burns, and there was no change in the localization of pigment particles, even for iron oxide black, which is a ferromagnetic substance. This suggests that even with static magnetic fields as strong as 9.4 T, the impact by its brief exposure was limited.

This study has four limitations. Firstly, we used one mouse for each pigment, so the sample size is small. However, we think these findings could potentially contribute to the current knowledge on this topic. Secondly, the temperature increase caused by the MRI may differ based on the tattoo shape. We intended for the circles to be the same as the areola diameter of 25-mm size in this study. We think it is necessary to evaluate the differences by shape and size in the future.

Thirdly, we performed the MRI the day after tattooing. Because there was erosion by tattoo, we could not evaluate whether erosion occurs by the MRI. However, due to the temperature change and histological change, we believe that the risk of erosion is nevertheless very low.

Finally, our study used ultra-high-field 9.4-T MRI, one of the highest MRI strengths in eastern Japan. We selected this device, which is too strong for clinical use, to inflict a static magnetic field and RF pulse sufficiently strong to elicit a response. However, resonance circuits and antenna effects differ from the strength of the magnetic field; therefore, additional evaluation using 1.5-T and 3.0-T MRI is required for a full exploration of the issue.

### Conclusions

Tattoo pigments containing metal components were applied to hairless mice undergoing 9.4-T ultra-high field MRI examinations. The observed temperature changes, color tone changes, and histological changes in tattooed areas noted in the mouse models were not enough to adversely affect the human body.

## Article Information

### Conflicts of Interest

None

### Sources of Funding

This work was supported by JSPS KAKENHI grant number JP15K19820.

The funding source has no role in study design; in the collection, analysis and interpretation of data; in the writing of the report; and in the decision to submit the article for publication.

### Acknowledgement

We would like to thank Prof. Masayuki Yokoyama and Dr. Koichi Shiraishi from medical engineering laboratory, research center for medical science, Jikei university school of medicine for the technical supports. Furthermore, we would like to thank Editage (www.editage.jp) for English language editing.

### Author Contributions

Shoichi Tomita: Corresponding author.

Takeshi Miyawaki: co-author.

### Ethical Statement

The protocol for the animal experiments was reviewed and approved by the Institutional Animal Care and Use Committee of the Jikei University (No. 2015-053) and conformed to the Guidelines for the Proper Conduct of Animal Experiments of the Science Council of Japan (2006).
